# *Culicidae *diversity, malaria transmission and insecticide resistance alleles in malaria vectors in Ouidah-Kpomasse-Tori district from Benin (West Africa): A pre-intervention study

**DOI:** 10.1186/1756-3305-3-83

**Published:** 2010-09-06

**Authors:** Armel Djènontin, Sahabi Bio-Bangana, Nicolas Moiroux, Marie-Claire Henry, Olayidé Bousari, Joseph Chabi, Razaki Ossè, Sébastien Koudénoukpo, Vincent Corbel, Martin Akogbéto, Fabrice Chandre

**Affiliations:** 1 Centre de Recherche Entomologique de Cotonou (CREC), 06 BP 2604 Cotonou, Bénin; 2 Institut de Recherche pour le Développement (IRD/UR016), 01 BP 4414 Cotonou, Bénin; 3 Laboratoire de lutte contre les Insectes Nuisibles (LIN/IRD), 911 Ave Agropolis BP 64501, 34394 Montpellier Cedex 5, France; 4 Service de Coopération française, Ambassade de France, Cotonou, Bénin

## Abstract

**Background:**

To implement an Insecticide Resistance Management (IRM) strategy through a randomized controlled trial (phase III), 28 villages were selected in southern Benin. No recent entomological data being available in these villages, entomological surveys were performed between October 2007 and May 2008, before vector control strategies implementation, to establish baseline data.

**Methods:**

Mosquitoes were sampled by human landing collection (16 person-nights per village per survey per village) during 5 surveys. Mosquitoes were identified morphologically and by molecular methods. The *Plasmodium falciparum *circumsporozoïte indexes were measured by ELISA, and the entomological inoculation rates (EIRs) were calculated. Molecular detection of pyrethroid knock down resistance (*Kdr*) and of insensitive acetylcholinesterase were performed.

**Results:**

44,693 mosquitoes belonging to 28 different species were caught from October 2007 to May 2008. Among mosquitoes caught, 318 were *An. gambiae s.s*., 2 were *An. nili*, 568 were *An. funestus s.s*., and one individual was *An. leesoni*. EIR was 2.05 ± 1.28 infective bites per human per 100 nights on average, of which 0.67 ± 0.60 were from *An. funestus *and 1.38 ± 0.94 infective bites were from *An. gambiae*. Important variations were noted between villages considering mosquito density and malaria transmission indicating a spatial heterogeneity in the study area. The *kdr *allelic frequency was 28.86% in *An. gambiae s.s*. on average and significantly increases from October 2007 (10.26%) to May 2008 (33.87%) in M molecular form of *An. gambiae s.s*. *Ace 1 *mutation was found in S molecular of *An. gambiae s.s *at a low frequency (< 1%).

**Conclusion:**

This study updates information on mosquito diversity and malaria risk in rural villages from south Benin. It showed a high spatial heterogeneity in mosquito distribution and malaria transmission and underlines the need of further investigations of biological, ecological, and behavioral traits of malaria vectors species and forms. This study is a necessary prerequisite to cartography malaria risk and to improve vector control operations in southern Benin.

## Background

Malaria remains a major cause of morbidity and mortality in sub-Saharan Africa and represents one of the most critical public health challenges for Africa. In 2008, 243 million cases of malaria was estimated worldwide leading to 863 000 deaths of which 89% were in the African Region [1]. Treatment with Artemisinin Combination Therapy (ACT), the use of Indoor Residual Spraying (IRS) and Insecticide Treated Nets (ITNs) represent the main approaches of malaria control [1]. Household ITNs ownership reached more than 50% in several high burden African countries [1]. Pyrethroids are the only insecticides used for net impregnation because of their strong efficacy, their fast acting effect at low doses and their low toxicity for mammals [2]. Unfortunately, pyrethroids resistance in malaria vectors has spread across Africa and is now present in most of countries where national malaria control programmes (NMCP) are implementing large scale distribution of Long Lasting Nets to populations at risk, i.e. children under five and pregnant women [3]. Up to now, there is no evidence that pyrethroids resistance reduce the effectiveness of ITNs for malaria control at operational scale [4]. However, a small scale field trial carried out in an area of resistance in southern Benin (Ladji) and Burkina Faso (Kou Valley) showed a reduction of personal protection and overall insecticidal effect of ITNs in experimental huts [5-7].

It is then urgent to find ways to manage this resistance in malaria vectors. In this context, malaria vectors control and insecticide resistance management tools based on the use (alone or in combination with pyrethroid-treated mosquito nets) of alternative classes of insecticides with different mode of action than pyrethroids were developed and have already been evaluated in experimental huts, with good results against wild populations of strongly resistant *An. gambiae *[6,7].

To validate these strategies, their impact on malaria transmission and insecticide resistance dynamic in malaria vectors must be accessed through randomized controlled trials under phase III. For that purpose, 28 villages were selected in Ouidah-Kpomasse-Tori (OKT) health district in southern Benin. No entomological data from OKT health district was available. Recent data relative to *Culicidae *fauna of Benin go back up to 1950s [8,9]. Thus, in order to collect baseline data relative to mosquito's diversity and abundance, malaria transmission and the prevalence of insecticide resistance alleles in malaria vectors, entomological surveys were performed between October 2007 and May 2008 in the study area before implementing the vector control strategies. This paper reports these baseline data.

## Methods

### Study area

**Figure 1:**
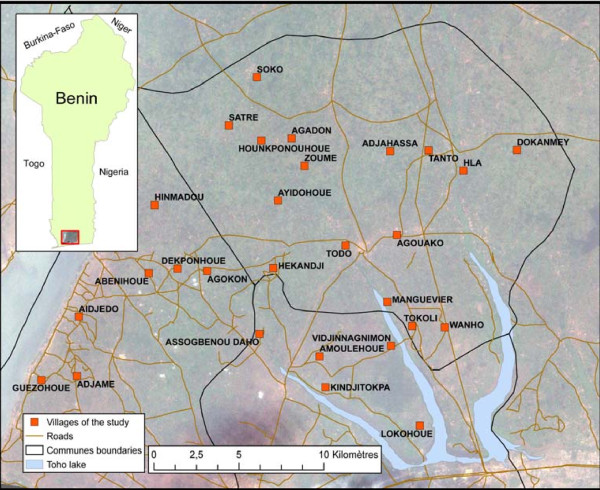
**Map of the study area**.

The study was carried out in OKT health district. Twenty eight villages were selected considering a size between 250-500 inhabitants, a distance between two villages higher than two kilometers and the absence of a local health center. The OKT health district is one of 36 health districts in Benin. This district has essentially a sub-equatorial climate, with two dry seasons (August-September and December-March), and two rainy seasons (April-July and October-November). The average annual rainfall is around 1200 mm, of which 700-800 mm come in the first rainy season and 400-500 mm come in the second rainy season. The average monthly temperatures vary between 27 and 31°C. The northern part of the health district is made of a plateau that drops into the Couffo valley and the Allada depression. The southern is watered by several ramification arms of Toho Lake (Fig 1). The study zone is totally cleared of the original equatorial forest. Currently, the vegetation is characterized by bushes and isolated trees, associated with areas with more or less densely populated areas with oil palm trees.

### Mosquito's collection and identification

Sampling of mosquitoes was done in 28 villages by human landing catches using tubes that were plugged with cotton. Mosquitoes collection was carried out during 5 surveys from October 2007 to May 2008 (2 in the beginning of rainy periods and 3 in dry periods) every 6 weeks both indoors and outdoors at 4 sites per village from 10 p.m. to 6 a.m. and for two consecutive nights per survey (i.e. 16 person-nights per village per survey). Teams of collectors were rotated among the collection points on different collection nights to minimize sampling bias. Ethical clearance was given for the study by the National Ethical Committee in Benin (Comité National Provisoire d'Ethique pour la Recherche en Santé) and IRD ethical committee (Comité Consultatif de Déontologie et d'Ethique). Collectors gave prior informed consent and they were vaccinated against yellow fever. Since study was done in area where malaria is endemic, adult collectors that already acquired immunity against malaria parasites, did not received chemoprophylaxis, but were medically supervised by local physicians in case of illness.

Mosquitoes were identified on the field to species level using morphological criteria according to the identification keys [10-12]. All mosquitoes belonging to the *An. gambiae *complex or *An. funestus *group were stored in individual tubes with silica gel and preserved at -20°C in the laboratory.

### Determination of EIRs

Heads and thoraces of anopheline females were tested by Enzyme-Linked Immunosorbent Assay (ELISA) for detection of *P. falciparum *circumsporozoite protein (CSP), as previously described [13]. Samples having an optical density higher than 3 times the average of the optical density of negative control were considered as positive. Those having an optical density between 2 and 3 times this average were considered as doubtful and were retested.

The CSP index was calculated as the proportion of mosquitoes found to be positive for CSP. Entomological inoculation rates (EIRs) were estimated as the number of infective bites per human per 100 nights.

### Molecular analysis

All mosquitoes belonging to *An. gambiae *complex and *Anopheles funestus *group were identified by polymerase chain reaction (PCR) at the species levels [14,15]. Molecular forms in *Anopheles gambiae s.s*. were also identified [16].

Molecular characterizations of the *Kdr *(west-African Leu-Phe mutation) and *Ace1 *mutations were carried out on all mosquitoes belonging to *An. gambiae *complex as previously described [17,18]

### Statistical analysis

Chi-square tests were done to determine the odds of association of the CSP index with malaria vectors *An. gambiae *and *An. funestus*.

Data relative to mosquito's density and EIRs according to villages were analyzed using a Linear Mixed Effects (LME) model implemented in R software and written as follow:

Log (x) = β_0 _+ β_1i _+ a_i_, where:

x = means of mosquitoes number (or EIRs) per village per survey

ß_0 _= estimated value of coefficient associated to the reference village

ß_1i _= estimated value of coefficient associated to a village i

a_i _= intra-village variance

i = village index

Villages were grouped according to their homogeneity for the entomological indicator considered taking into account ß_i _and its P value. In each group, the average of the number of mosquitoes caught per village per survey (or the number of infective bites per human per 100 nights) was calculated with its 95% confidence interval.

The genotypic differentiation of *kdr *and *Ace1 *loci was tested using the Fischer exact test implemented in GenePop software [19].

## Results

### Species diversity and density

A total of 44,693 mosquitoes belonging to 28 species were caught (table 1). *Mansonia Africana *(44.84%), *Culex gr. decens *(29.91%), *Culex quinquefasciatus *(9.81%), and *Culex nebulosis *(6.22%) were the most abundant species caught. The proportion of malaria vectors (*Anopheles gambiae *and *Anopheles funestus*) was very low (~2%).

**Table 1:** Mosquitoes species caught from October 2007 to May 2008 in the study area

Species	Indoor	Outdoor	Total	%
*Aedes aegypti*	388	318	706	1.58
*Aedes gr. palpalis*	12	28	40	0.09
*Aedes gr. tarsalis*	4	4	8	0.02
*Aedes luteocephalus*	21	41	62	0.14
*Aedes sp*	13	37	50	0.11
*Aedes vittatus*	199	396	595	1.33
*Anopheles brohieri*	1	0	1	0.00
*Anopheles coustani*	3	5	8	0.02
*Anopheles funestus*	314	265	579	1.30
*Anopheles gambiae*	149	173	322	0.72
*Anopheles nili*	1	1	2	0.00
*Anopheles pharoensis*	60	135	195	0.44
*Anopheles ziemanni*	12	20	32	0.07
*Culex annulioris*	185	428	613	1.37
*Culex duttoni*	36	18	54	0.12
*Culex fatigans*	270	436	706	1.58
*Culex gr. decens*	4720	8649	13369	29.91
*Culex nebulosis*	1040	1738	2778	6.22
*Culex perfuscus*	2	0	2	0.00
*Culex poicilipes*	2	14	16	0.04
*Culex quinquefasciatus*	1170	3213	4383	9.81
*Culex tigripes*	17	38	55	0.12
*Cx sp*	17	17	34	0.08
*Cx thalassius*	10	16	26	0.06
*Eretmapodites gr. quinquevittatus*	0	3	3	0.01
*Mansonia africana*	7652	12390	20042	44.84
*Mansonia uniformis*	7	4	11	0.02
*Uranotaenia gr bilineata*	1	0	1	0.00

Total	16306	28387	44693	

All mosquitoes belonging to *Anopheles gambiae s.l*., were *An. gambiae s.s*. (217 from S molecular form and 101 from M form). Concerning *Anopheles funestus *group all mosquitoes were identified by PCR as *Anopheles funestus s.s*. except one individual which was *An. leesoni*, caught in Tokoli-Vidjinangnimon village.

Large variations were observed between villages considering mosquito's density. An average of 212 ± 46 *Culicidae *per village per survey and 531 ± 139 *Culicidae *per village per survey were caught in two different homogenous villages groups (Table 2). Concerning *An. gambiae s.l*. on average 0.4 ± 0.2; 1.8 ± 0.6 and 7.4 ± 3.5 individuals were caught per village per survey in three different homogenous villages groups (Table 3). In villages close to a arm of the Toho Lake, 13.48 ± 5.9 *An. funestus s.l*. were caught on average per village per survey, in contrast with 0.29 ± 0.1 in the others villages (Table 4).

**Table 2:** Average of *Culicidae *number caught per survey per village from October 2007 to May 2008 with 95% confidence intervals

Villages groups	Average *Culicidea *number per survey per village with 95% confidence intervals
**Group 1: **Agouako; Assogbenoudaho; Guezohoue; Hla; Todo; Zoume; Adjahassa; Satre; Agadon; Aidjedo; Ayidohoue; Dokanme; Tokoli Vidjinangnimon; Hekandji; Hounkponouhoue; Dekponhoue; Soko; Wanho; Tokoli; Tanto	212 ± 46
**Group 2 **: Lokohoue; Tokoli Vidjinangnimon; Manguevier; Adjame-Allagbede; Amoulehoue; Kindjitokpa; Abenihoue; Hinmandou	531 ± 139

**Table 3:** Average of *An. gambiae s.l*. number caught per survey per village from October 2007 to May 2008 with 95% confidence intervals

Villages groups	Average *An. gambiae s.l*. number per survey per village with 95% confidence intervals
**Group 1: **Adjame-Allagbede; Agouako; Assogbenoudaho; Kindjitokpa; Guezohoue; Hinmandou; Hla; Abenihoue; Todo; Zoume	0.4 ± 0.2
**Group 2**: Adjahassa; Satre; Lokohoue; Agadon; Aidjedo; Ayidohoue; Dokanme; Tokoli Vidjinangnimon; Hekandji; Agokon; Hounkponouhoue; Dekponhoue; Manguevier	1.8 ± 0.6
**Group 3**: Amoulehoue; Soko; Tokoli; Tanto; Wanho	7.4 ± 3.5

**Table 4:** Average of *An. funestus s.l*. number caught per survey per village from October 2007 to May 2008 with 95% confidence intervals

Villages groups	Average *An. funestus s.l*. number per survey per village with 95% confidence intervals
**Group 1**: Adjame-Allagbede; Agouako; Assogbenoudaho; Guezohoue; Hinmandou; Hla; Abenihoue; Todo; Zoume; Adjahassa; Satre; Agadon; Agokon; Aidjedo; Ayidohoue; Dokanme; Hekandji; Hounkponouhoue; Dekponhoue; Soko	0.29 ± 0.1
**Group 2**: Amoulehoue; Tokoli; Tanto; Lokohoue; Manguevier; Kindjitokpa; Tokoli Vidjinangnimon; Wanho	13.48 ± 5.9

### Vectors infection to CSP and malaria transmission risk

The CSP positivity rate was 9.63 ± 3.2% in *An. gambiae s.s*. (5.94 ± 0.4% in M molecular form and 11.52 ± 0.2% in S form) and 2.64 ± 1.3% in *An. funestus s.l*.. *An. gambiae s.s*. was more infected than *An. funestus s.l*. (OR = 4.00 (95%IC 2.13-7.54), P < 0.001). The average of EIR from October 2007 to May 2008 was 2.05 ± 1.28 infective bites per human per 100 nights of which 0.67 ± 0.60 infective bites of *An. funestus *per human per 100 nights and 1.38 ± 0.94 infective bites of *An. gambiae *per human per 100 nights. EIR was very variable according to villages. Two homogenous villages groups with respectively 0.7 ± 0.4 and 6.1 ± 3.4 infective bites per human per 100 nights were observed (Table 5).

**Table 5:** Number of infective bites per human per 100 nights from October 2007 to May 2008 with 95% confidence intervals

Villages groups	Number of infective bites per human per 100 nights with 95% confidence intervals
**Group 1 **: Adjame; Agouako; Aidjedo; Asogbenoudaho; Ayidohoue; Adjahassa; Dokanme; Kindjitokpa; Guezohoue; Hinmandou; Hla; Hounkponouhoue; Abenihoue; Dekponhoue; Manguevier; Satre; Todo; Tokoli; Wanho; Agadon; Zoume	0.7 ± 0.4
**Group 2 **: Amoulehoue; Tokoli Vidjinangnimon; Hekandji; Agokon; Soko; Lokohoue; Tanto	6.1 ± 3.4

### *Kdr *resistance gene status in *An. gambiae*.

The average of *kdr *allelic frequency from October 2007 to May 2008 was 28.86% in *An. gambiae s.s*. At the beginning of the study (From October to December 2007), *kdr *allelic frequency was significantly higher in S molecular form of *An. gambiae s.s*. (28.08%) compared to the M molecular form (10.26%) (P = 0.001). This allelic frequency significantly increased in the M molecular form and has reached 33.87% (P < 0.001) from January to May 2008 (Table 6).

**Table 6:** kdr in M and S molecular forms of *An. gambiae *s.s. from October 2007 to May 2008 in the study area

	October-December 2007		January-May 2008	
	
	M form	S form	M form	S form
RR	0	10	13	16
RS	8	62	16	20
SS	31	74	33	35
Total	39	146	62	71
Allelic frequency (%)	**10.26**^a^	**28.08**^b^	**33.87**^b^	**36.62**^b^

*Kdr *allelic frequency values carrying the same letter was not significantly different (P > 0.05)

### Insensitive acetylcholinesterase gene status in *An. gambiae s.l*

The allelic frequency of this gene was less than 1%. Only two heterozygous individuals was found in the S molecular form of *An. gambiae s.s*. at Tanto and Hekandji during October 2007.

## Discussion

The present study provides entomological baseline data on OKT health district. Twenty eight different species were caught during surveys whereas Huttel (1950) and Hamon (1954) had collected respectively 13 species (adult's collection in the dwellings) and 45 species (adult and larval collection) in the south-east of Benin (Cotonou and Porto-Novo) [8,9]. If the *Culicidae *diversity is lower in our study, we have identified 3 *Anopheles *species that was not mentioned in Hamon study: *An. brohieri*, *An. coustani *and *An. nili*. These 3 species were present at very low densities, explaining why they were not reported previously. More unexpected is the predominance in our study of *Mansonia africana *and the presence of *Cx quinquefasciatus *that were not reported by Hamon. The relative abundance of *Cx. quinquefasciatus *is particularly surprising for a species which is adapted to polluted larval breeding sites and usually more familiar with urban environment than traditional villages from this study. Results showed a high heterogeneity of the study area in terms of *Culicidae *and especially malaria vectors abundance. It could be due to the geographic pattern of this area that is characterized by a northern part made of a plateau and the southern watered by several ramification arms of Toho Lake. *An. funestus *density was higher in villages close to arms of Toho Lake (< 2 km), since some of its larval breeding sites are the bank of lake or river with vegetation. In most villages of the study area, the soil is made of a thick bed of a mixture of sand and clay allowing a rapid water infiltration after rain. This impedes the formation of *An. gambiae *larval breeding sites that could explain the low density of malaria vectors. However, on the 5 surveys conducted in each village, only two were conducted during a rainy period (in the beginning of the rainy periods). This could explain the very low number of anopheles vectors caught during the study. Further studies conducted on a long period and taking into account environmental factors are required to better understand mosquito's distribution determinants in this area.

*Anopheles gambiae s.s*. and *An. funestus s.s*. were shown to be the main vectors in the study area, confirming previous studies in West Africa [20-22]. The relative abundance of *An. funestus *in the study area and its CSP positivity rate (2.64 ± 1.3) indicate that this mosquito is involved in malaria transmission in southern Benin. No data relative to resistance status and mechanisms of this vector being available in Benin, further studies about *An. funestus *populations and their resistance mechanisms are required. The EIR, 2.05 ± 1.28 infective bites per human per 100 nights on average, indicates that in the study area, malaria is mesoendemic. These data agree with parasitological and clinical data collected in the same way in these villages indicating an annual prevalence rate of 21.8% (19.1-24.4) in young asymptomatic children and a clinical incidence of *Plasmodium falciparum *malaria of 1.5 (1.2-1.9) per child per year (Damien *et al*. 2010, unpublished data).

The *kdr-w *resistance allele was identified in *An. gambiae *populations examined in this study and have significantly increased from October 2007 to May 2008 in M molecular form in contrast with S form, indicating probably that M and S molecular forms of this vector undergo different selection pressures in the study area and underlines the need of further studies taking into account ecological and others factors. The presence of the *kdr-w *allele in *An. gambiae s.s*. and the increase of its frequency are worrying because this gene and metabolic resistance mechanisms in southern Benin appears to have a significant impact on LLIN efficacy as demonstrated by recent experimental hut trial [23]. Many other studies have showed a strong decrease of Pyrethroid-Treated Nets performance against pyrethroid-resistant malaria vectors in Benin [5,6], Burkina Faso [7], and Côte d'Ivoire [24].

Regarding carbamate resistance, we confirmed the low allelic frequency of the a*ce-1^R ^*allele (< 1%) in *An. gambiae s.s*. in West Africa [25], showing that there is no (or very low) selection pressure on this allele in the study area. However, a monitoring of this resistance gene is required considering the large scale implementation of Indoor Residual Spraying using bendiocarb by President's Malaria Initiative program in south Benin that will inevitably increase the selection pressure on vectors populations.

## Conclusion

This study updates information on mosquito's distribution and their role in malaria transmission in southern Benin. It showed a high spatial heterogeneity in mosquitoes distribution and malaria transmission and then underlines the need of further investigations of biological, ecological, and behavioral traits of malaria vectors species and forms. That will allow to cartography malaria risk and then to improve vector control interventions in Benin.

## Competing interests

The authors declare that they have no competing interests.

## Authors' contributions

FC and M-CH conceived of the study. AD, JC and FC have participated in the design of the study. Entomologic data was collected by AD, JC, RO, SK. AD, SK and RO carried laboratory analysis. AD, FC, OB and VC have participated in the analysis and interpretation of data. Maps were provided by SBB and NM. The manuscript has been drafted by AD. AD, SBB, NM, M-CH, OB, JC, RO, SK, VC, MA and FC have been involved in manuscript revising. All authors read and approved the final manuscript.

## References

[B1] WHO World Malaria Report 2009 2009 World Health Organzation, Geneva

[B2] Zaim M Aitio A Nakashima N Safety of pyrethroid-treated mosquito nets Med Vet Entomol 2000 14 1 5 10.1046/j.1365-2915.2000.00211.x 10759305

[B3] Santolamazza F Calzetta M Etang J Barrese E Dia I Caccone A Donnelly MJ Petrarca V Simard F Pinto J della Torre A Distribution of knock-down resistance mutations in *Anopheles gambiae *molecular forms in west and west-central Africa Malar J 2008 7 74 10.1186/1475-2875-7-74 18445265 PMC2405802

[B4] Henry MC Assi SB Rogier C Dossou-Yovo J Chandre F Guillet P Carnevale P Protective efficacy of lambda-cyhalothrin treated nets in *Anopheles gambiae *pyrethroid resistance areas of Cote d'Ivoire Am J Trop Med Hyg 2005 73 859 864 16282294

[B5] N'Guessan R Corbel V Akogbéto M Rowland M Reduced efficacy of insecticide-treated nets and indoor residual spraying for malaria control in pyrethroid resistance area, Benin Emerg Infect Dis 2007 13 199 206 10.3201/eid1302.060631 17479880 PMC2725864

[B6] Djènontin A Chabi J Baldet T Irish S Pennetier C Hougard JM Corbel V Akogbéto M Chandre F Managing insecticide resistance in malaria vectors by combining carbamate-treated plastic wall sheeting and pyrethroid-treated bed nets Malar J 2009 8 233 10.1186/1475-2875-8-233 19843332 PMC2776024

[B7] Djènontin A Chandre F Dabiré KR Chabi J N'Guessan R Baldet T Akogbéto M Corbel V The Indoor Use of Plastic Sheeting Impregnated with Carbamate in Combination with Long Lasting Insecticidal Mosquito Nets for the Control of Pyrethroid-resistant Malaria Am J Trop Med Hyg 2010 83 266 270 10.4269/ajtmh.2010.10-0012 20682865 PMC2911168

[B8] Huttel J Note sur la répartition des moustiques dans le Bas-Dahomey Bull Sot Path Exot 1950 43 563 566

[B9] Hamon J Contribution à l'étude des Culicidés de la région de Porto-Novo (Bas-Dahomey) Ann parasitologie 1954 29 588 594 14377156

[B10] Edwards F Mosquitoes of the Ethiopian Region III. Culicine adults and pupae British Museum (Nat Hist), London 1941

[B11] Gillies MT De Meillon BL The Anophelinae of Africa south of the Sahara 1968 Publication of the South African Institute for Medical Research, Johannesburg 54

[B12] Gillies MT Coetzee M A Supplement to the Anophelinae of Africa South of the Sahara (Afrotropical Region) 1987 Publication of the South African Institute for Medical Research, Johannesburg 55

[B13] Wirtz RA Zavala F Charoenvit Y Campbell GH Burkot T Schneider I Esser KM Beaudoin RL Andre RG Comparative testing of monoclonal antibodies against *Plasmodium falciparum *sporozoites for ELISA development Bull World Health Organ 1987 65 39 45 3555879 PMC2490858

[B14] Scott JA Brogdon WG Collins FH Identification of single specimens of the *Anopheles gambiae *complex by the polymerase chain reaction Am J Trop Med Hyg 1993 49 520 529 8214283 10.4269/ajtmh.1993.49.520

[B15] Koekemoer LL Kamau L Hunt RH Coetzee M A cocktail polymerase chain reaction assay to identify members of the *Anopheles funestus *(Diptera: Culicidae) group Am J Trop Med Hyg 2002 66 804 811 12224596 10.4269/ajtmh.2002.66.804

[B16] Favia G Lanfrancotti A Spanos L Siden Kiamos I Louis C Molecular characterization of ribosomal DNA polymorphisms discriminating among chromosomal forms of *Anopheles gambiae s.s* Insect Mol Biol 2001 10 19 23 10.1046/j.1365-2583.2001.00236.x 11240633

[B17] Martinez-Torres D Chandre F Williamson MS Darriet F Berge JB Devonshire AL Guillet P Pasteur N Pauron D Molecular characterization of pyrethroid knockdown resistance (*kdr*) in the major malaria vector *Anopheles gambiae s.s* Insect Mol Biol 1998 7 179 184 10.1046/j.1365-2583.1998.72062.x 9535162

[B18] Weill M Malcolm C Chandre F Mogensen K Berthomieu A Marquine M Raymond M The unique mutation in ace-1 giving high insecticide resistance is easily detectable in mosquito vectors Insect Mol Biol 2004 13 1 7 10.1111/j.1365-2583.2004.00452.x 14728661

[B19] Raymond M Rousset F GENEPOP (version 1.2): population genetics software for exact tests and ecumenicism J Heredity 1995 86 248 249

[B20] Akogbéto M Entomological study on the malaria transmission in coastal and lagoon areas: the case of a village built on a brackish lake Ann Soc Belg Med Trop 1995 75 219 227 8849299

[B21] Fontenille D Simard F Unravelling complexities in human malaria transmission dynamics in Africa through a comprehensive knowledge of vector populations Comp Immunol Microbiol Infect Dis 2004 27 357 375 10.1016/j.cimid.2004.03.005 15225985

[B22] Louise A Kelly-Hope L Ellis McKenzie F The multiplicity of malaria transmission: a review of entomological inoculation rate measurements and methods across sub-Saharan Africa Malar J 2009 8 19 10.1186/1475-2875-8-19 19166589 PMC2656515

[B23] WHO Report of the twelfth WHOPES Working group meeting WHO/HTM/NTD/WHOPES 2009 1

[B24] N'Guessan R Darriet F Doannio JM Chandre F Carnevale P Olyset Net efficacy against pyrethroid-resistant *Anopheles gambiae *and *Culex quinquefasciatus *after 3 years' field use in Côte d'Ivoire Med Vet Entomol 2001 15 97 104 10.1046/j.1365-2915.2001.00284.x 11297108

[B25] Djogbénou L Dabire R Diabate A Kengne P Akogbéto M Hougard JM Chandre F Identification and geographic distribution of the ACE-1R mutation in the malaria vector *Anopheles gambiae *in south-western Burkina Faso, West Africa Am J Trop Med Hyg 2008 78 298 302 18256433

